# Pineal-dependent increase of hypothalamic neurogenesis contributes to the timing of seasonal reproduction in sheep

**DOI:** 10.1038/s41598-018-24381-4

**Published:** 2018-04-18

**Authors:** Martine Batailler, Didier Chesneau, Laura Derouet, Lucile Butruille, Stéphanie Segura, Juliette Cognié, Joëlle Dupont, Delphine Pillon, Martine Migaud

**Affiliations:** 1 INRA, UMR 85 Physiologie de la Reproduction et des Comportements, F-37380 Nouzilly, France; 2 CNRS, UMR7247, F-37380 Nouzilly, France; 3 Université de Tours, F-37041 Tours, France; 4 Institut Français du Cheval et de l’Equitation (IFCE), F-37380 Nouzilly, France

## Abstract

To survive in temperate latitudes, species rely on the photoperiod to synchronize their physiological functions, including reproduction, with the predictable changes in the environment. In sheep, exposure to decreasing day length reactivates the hypothalamo-pituitary-gonadal axis, while during increasing day length, animals enter a period of sexual rest. Neural stem cells have been detected in the sheep hypothalamus and hypothalamic neurogenesis was found to respond to the photoperiod. However, the physiological relevance of this seasonal adult neurogenesis is still unexplored. This longitudinal study, therefore aimed to thoroughly characterize photoperiod-stimulated neurogenesis and to investigate whether the hypothalamic adult born-cells were involved in the seasonal timing of reproduction. Results showed that time course of cell proliferation reached a peak in the middle of the period of sexual activity, corresponding to decreasing day length period. This enhancement was suppressed when animals were deprived of seasonal time cues by pinealectomy, suggesting a role of melatonin in the seasonal regulation of cell proliferation. Furthermore, when the mitotic blocker cytosine-b-D-arabinofuranoside was administered centrally, the timing of seasonal reproduction was affected. Overall, our findings link the cyclic increase in hypothalamic neurogenesis to seasonal reproduction and suggest that photoperiod-regulated hypothalamic neurogenesis plays a substantial role in seasonal reproductive physiology.

## Introduction

In order to cope with the harsh annual changes of their environment, species living in temperate and arctic latitudes use a strategy relying on annual changes in day length or photoperiod to synchronize their physiological functions including reproduction with the constraints of external climatic and food availability^1^. In seasonal vertebrates, gonadotropin-releasing hormone (GnRH) neurons are activated annually to trigger pituitary gonadotropins secretion production, which consecutively activates the gonads. In sheep, this cyclic reactivation arises as day length decreases in the fall and extends to the beginning of winter, while for the rest of the year sheep enter a period of sexual rest. Photic information, responsible for synchronizing these neuroendocrine rhythms, is perceived by the retina and transmitted via a neuronal pathway to the suprachiasmatic nucleus. The suprachiasmatic nucleus then regulates the sympathetic innervation of the pineal gland which leads to melatonin being secreted at night^2^. Short photoperiod-dependent physiological changes can be initiated by administering exogenous melatonin under long-photoperiod exposure^3–6^, indicating that pineal melatonin plays a key role in these processes^7,8^.

Plasticity is an important feature of brain function since continuous adaptation to changing conditions is necessary to preserve homeostasis. Adult neurogenesis is a plasticity mechanism conserved throughout the animal kingdom. It relies on the existence of mitotically active neural stem cells (NSCs) located within highly specialized microenvironments called neurogenic niches. These specific areas include the sub-ventricular zone (SVZ) of the forebrain and the sub-granular zone (SGZ) of the hippocampal dentate gyrus^9^. Recently the hypothalamus, the central regulator of body homeostasis including reproduction, feeding, growth and metabolism, has emerged as a host site for such a neurogenic niche^10–13^. A growing number of studies shows that, in physiological conditions, the adult hypothalamus, specifically its most ventrocaudal part including the arcuate nucleus, has the capacity to proliferate and generate neurons in a constitutive manner^14,15^. We have recently revealed such a neurogenic niche located in the adult sheep hypothalamus^11,16^. Moreover, the number of dividing hypothalamic cells increased in December compared to July^17^, and this was independent of the ovarian steroid feedback^17^. This enhanced cell proliferation was shown to be linked to a robust increase in neurogenesis during decreasing day length demonstrated through immunohistochemical detection of doublecortin (DCX), a marker of newborn neurons^11,16,18^. However, these studies only evaluated proliferation at two time points in the year.

In the present study, we further assessed cell proliferation longitudinally in the three neurogenic niches of the adult ewe, namely the hypothalamus, the SGZ and the SVZ, in order to determine the proliferation pattern across the year. We next investigated whether the seasonal variations of proliferative activity observed in the hypothalamic and SGZ neurogenic niches were pineal dependent. Finally, we provided evidence that adult hypothalamic neurogenesis was linked functionally to the seasonal timing of reproduction.

## Results

### Time points of bromodeoxyuridine administration

**Figure 1:**
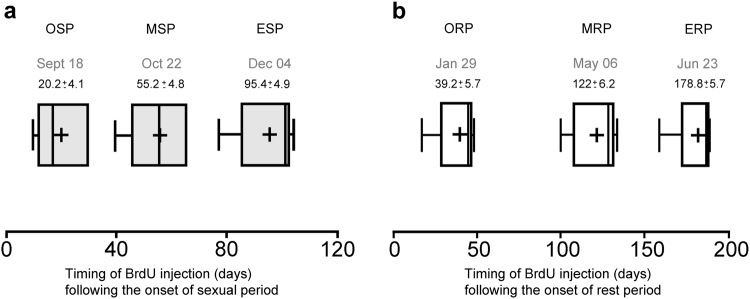
BrdU administration at six time points in the year. (**a** and **b**) Plots representing when BrdU was administered (20 mg/kg, single i.v. administration) during the sexual period (SP; a, grey plots) and during the rest period (RP; b, white plots); OSP: onset of the sexual period, MSP: middle of the sexual period, ESP: end of the sexual period, ORP: onset of the rest period, MRP: middle of the rest period, ERP: end of the rest period. Cyclic activity was determined using plasma progesterone concentration in samples collected twice weekly.

The decreasing day length of the fall extending up to the beginning of winter, corresponds to the sexual activity period (SP) that lasts for approximately 158 days in the Ile-de-France ewes raised at a latitude of 47°33′N (Nouzilly; France). On the other hand, the increasing day length corresponds to the anestrus- a period of sexual rest (RP), lasting for around 200 days in this breed^19^. In this study, first we determined the six time points of the year at which intravenous (i.v.) injections of the thymidine analogue Bromodeoxyuridine (BrdU) would be performed according to the reproductive status of the ewes assessed through plasma progesterone concentration. Thus, a first group of ewes received i.v. injections of BrdU at three time points in their sexual activity period (SP), on September 18, October 22, and December 4, corresponding to the onset of the SP (OSP): 20.2 ± 4.1, the middle of the SP (MSP): 55.2 ± 4.8 and the end of the SP (ESP): 95.4 ± 4.9 days after the onset of the SP respectively (Fig. 1a). Similarly, a second group of ewes were administered BrdU in their sexual rest period (RP) on January 29, May 6 and June 23, corresponding to the onset of the RP (ORP): 39.2 ± 5.7, the middle of the RP (MRP): 122 ± 6.2 and the end of the RP (ERP): 178.8 ± 5.7 days after the onset of the RP respectively (Fig. 1b).

### Hypothalamic cell proliferation follows a seasonal time course

**Figure 2:**
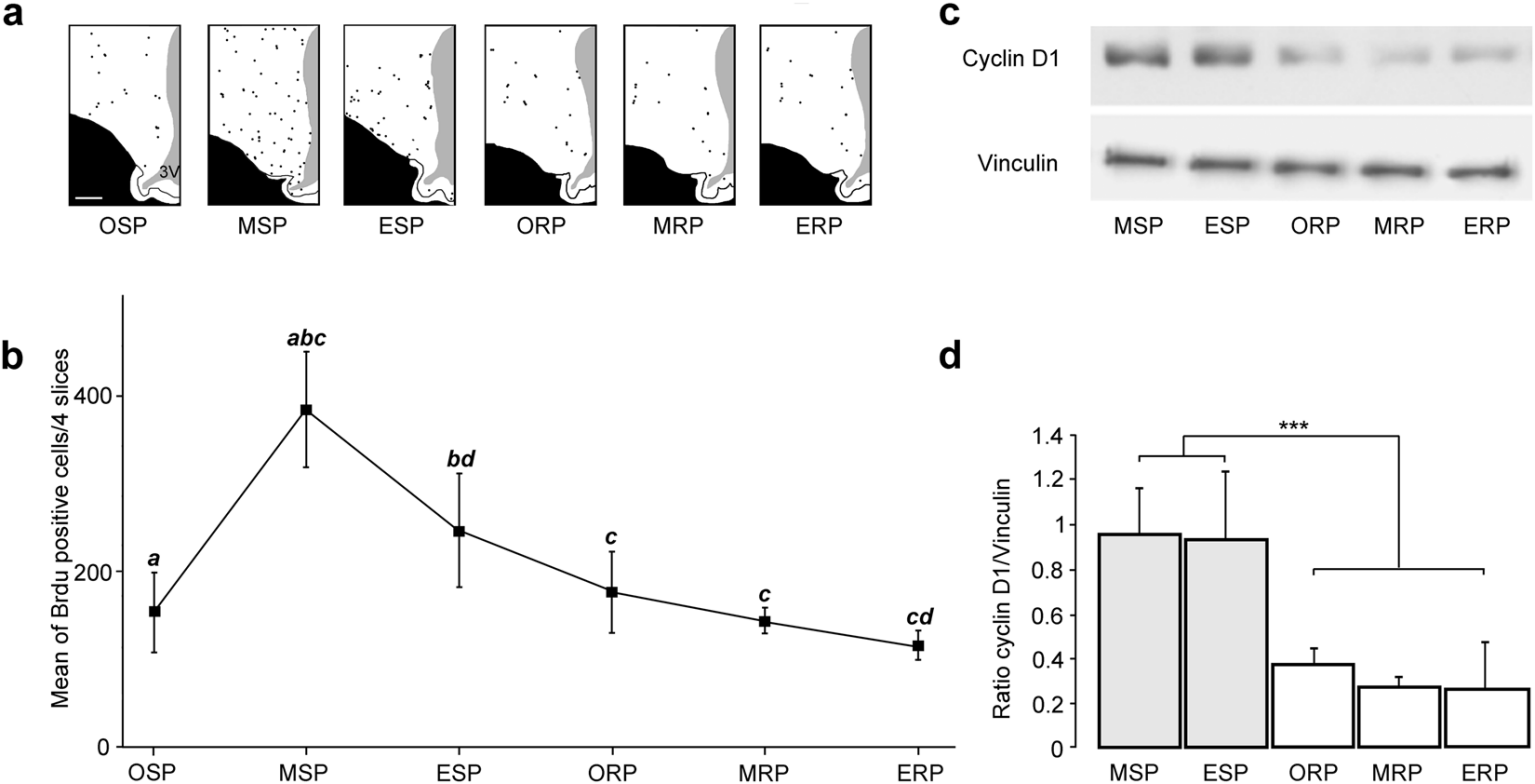
Quantification of longitudinal cell proliferation in sheep hypothalamus. (**a**) Representative schematic drawings of hypothalamic sections from each time point in the year, OSP, MSP, ESP, ORP, MRP and ERP. Each dot represents one BrdU + nucleus. 3 V = third ventricle. Scale bar: 1 mm. (**b**) Number of BrdU + nuclei at the MSP, ESP, ORP, MRP and ERP. Results are expressed as the mean number of BrdU + cells from four sections, error bars represent mean ± SEM (n = 5 animals per group). Different letters indicate significant differences at a and c: ***p < 0.001; b: **p < 0.01; d: *p < 0.05. (**c**) Kinetics of cyclin D1 expression measured by western blotting at the OSP, MSP, ESP, ORP, MRP and ERP time points. Samples contained equal protein concentrations, as confirmed following the reprobing of each membrane with an anti-vinculin antibody (n = 5 animals per group). (**d**) Cyclin D1 and vinculin immunoreactivity were quantified by scanning densitometry and the ratio cyclin D1/vinculin is shown. ***P < 0.001.

BrdU incorporated into proliferating cells was assessed using immunohistochemistry in coronal hypothalamic sections of the ewe brains. Figure 2a shows representative schematic drawings illustrating the spatial distribution of BrdU-positive nuclei in the hypothalami of ewes collected at six times in the year at 24 h post injection. A single peak of cell proliferation revealed by BrdU-positive nuclei, corresponding to the mean number of BrdU-positive cells, occurred in the middle (i.e.: at 55 days following onset) of the SP (MSP; Fig. 2b; 378.4 ± 25.7 BrdU + nuclei). A high level of cell proliferation was maintained up to the end of SP (ESP, Fig. 2b; 254.2 ± 22.9 BrdU + nuclei). At the onset of RP (ORP), cell proliferation dropped to a minimum and remained at a low level up to the end of RP (ERP, Fig. 2b; 216.6 ± 20.5, 173.2 ± 18.9 and 151.4 ± 13.5 BrdU + nuclei for ORP, MRP and ERP respectively). The expression of cyclin D1 estimated through the CyclinD1/vinculin ratio, which normalizes Cyclin D1 expression, was found significantly higher at MSP and ESP than at ORP, MRP and ESP (Fig. 2c and d, p < 0.001 for post hoc analysis).

### Neurogenic niches are differentially sensitive to photoperiod

**Figure 3:**
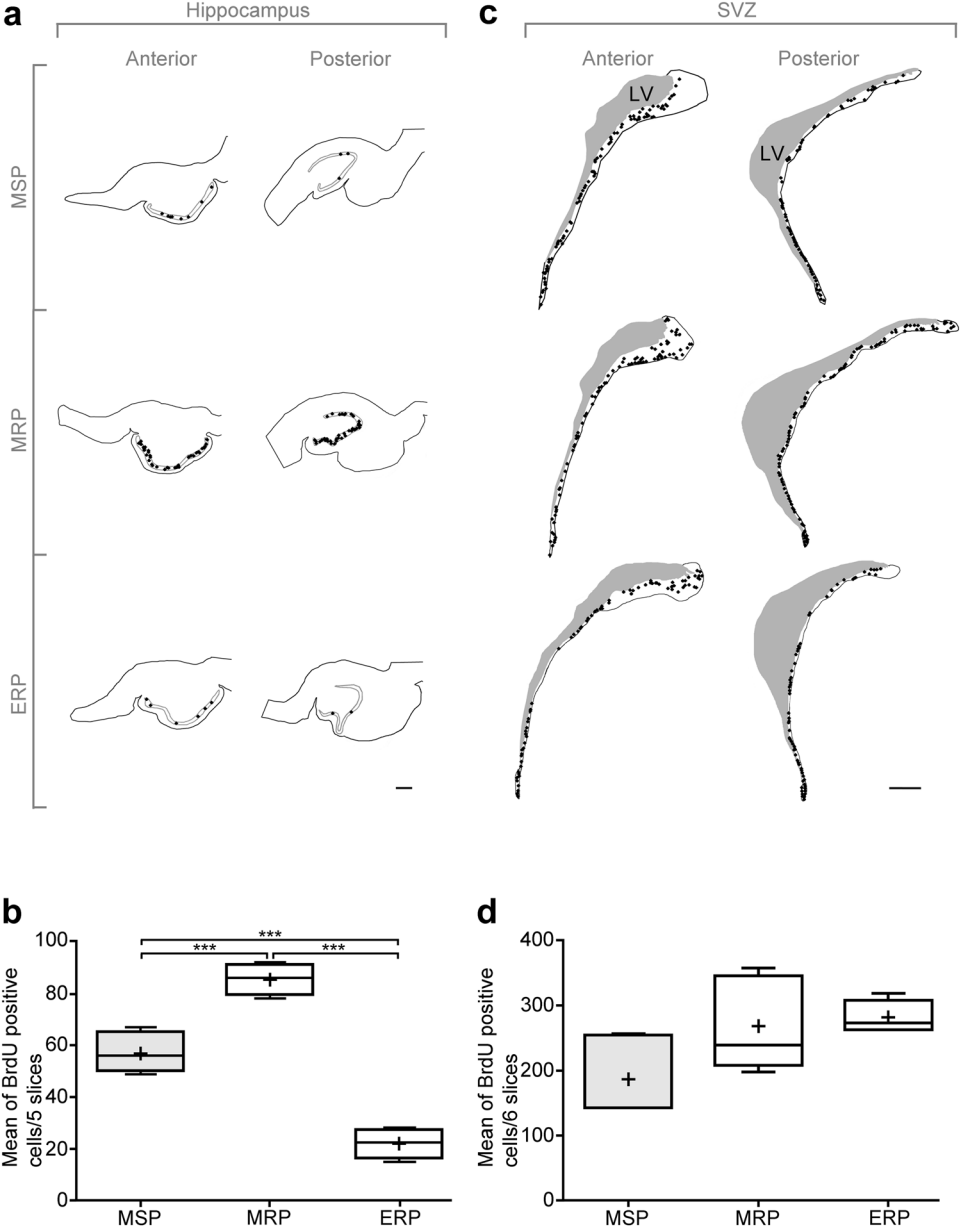
Region-specific effect of the season on cell proliferation in the dentate gyrus (**a**,**b**) and the subventricular zone (SVZ) of the lateral ventricle (**c**,**d**). (**a**) Representative schematic drawings of anterior (left panels) and posterior (right panels) hippocampal sections from MSP, MRP and ERP. Each dot represents one BrdU + nucleus. Scale bar: 1 mm. (**b**) Quantification of BrdU + nuclei at three time points of the year. Results are expressed as the mean number of BrdU + cells from 5 sections at MSP (grey plot), MRP and ERP (white plots). Error bars represent mean ± SEM (n = 5 animals per group). ***p < 0.001. (**c**) Representative schematic drawings of anterior (left panels) and posterior (right panels) SVZ sections from MSP, MRP and ERP. Each dot represents one BrdU + nucleus. LV: lateral ventricle. Scale bar: 1 mm. (**d**) Quantification of BrdU + nuclei at three time points in the year. Results are expressed as the mean number of BrdU + cells from 6 sections at MSP (grey plot), MRP and ERP (white plots). Error bars represent mean ± SEM (n = 5 animals per group).

Seasonal changes in neurogenesis in the SGZ and the SVZ have been documented in several photoperiodic rodent species^20–24^. Since no difference was observed between the anterior and posterior labeling (Fig. 3a), data were pooled for quantitative analysis. A peak of hippocampal cell proliferation measured as the mean number of BrdU-positive nuclei in five slices per animal (n = 6) was observed in the middle of the RP (MRP, Fig. 3a and b, p < 0.001). Moderate hippocampal cell proliferation was observed in the middle of the SP (MSP, Fig. 3a and b, p < 0.001), whereas the lowest number of BrdU positive nuclei was recorded at the end of RP (ERP, Fig. 3b, p < 0.001).

In contrast to what was observed in the SGZ and the hypothalamic neurogenic niche, cell proliferation in the SVZ was similar throughout the year thus showing no seasonal variation (Fig. 3c and d).

### Pinealectomy abolishes the increase in cell proliferation in the photoperiod sensitive neurogenic niches

**Figure 4:**
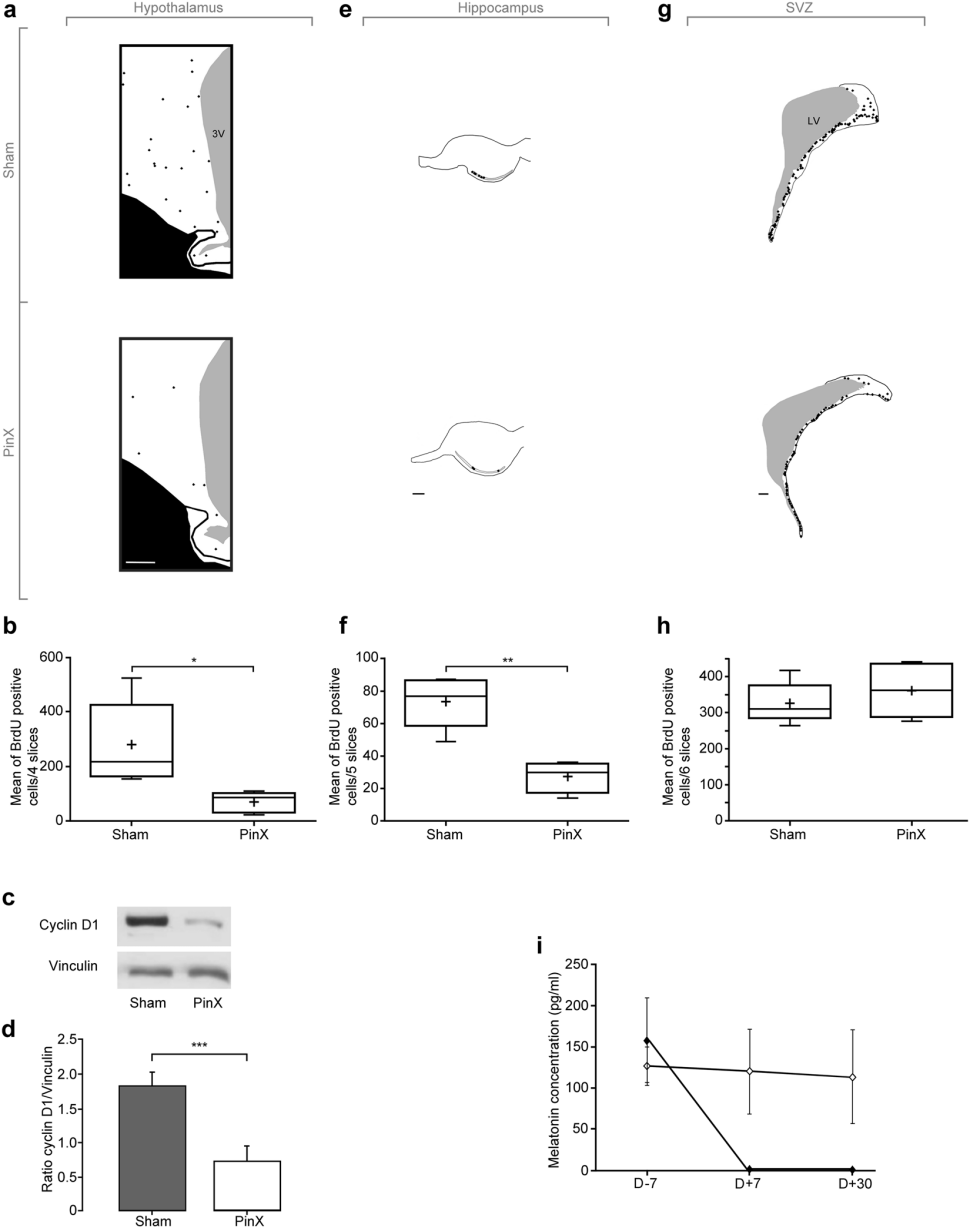
Pinealectomy affects cell proliferation in the hypothalamus (**a**–**d**) and in the dentate gyrus (**e**,**f**) but not in the SVZ (**g**,**h**). (**a**) Representative schematic drawings of hypothalamic sections from sham-operated (Sham, upper panel) and pinealectomized animals (PinX, lower panel). Each dot represents one BrdU + nucleus. 3 V: third ventricle. Scale bar: 1 mm. (**b**) Quantification of BrdU + nuclei in the hypothalamus of sham and PinX-operated animals. Results are expressed as the mean number of BrdU + cells from four sections. Experiments were performed during the sexual period (MRP). Error bars represent mean ± SEM (n = 5 animals per group). *p < 0.05. (**c**) Cyclin D1 expression was measured by western blotting in the hypothalamus of sham- and PinX-operated animals. Samples contained equal protein concentrations, as confirmed following the reprobing of each membrane with an anti-vinculin antibody. (**d**) Cyclin D1 and vinculin immunoreactivity were quantified by scanning densitometry and the ratio cyclin D1/vinculin ratio was represented (n = 5 animals per group). ***P < 0.001. (**e**) Representative schematic drawings of hippocampal sections from sham-operated (Sham, upper panel) and pinealectomized animals (PinX, lower panel). For each condition, anterior and posterior sections were shown. Each dot represents one BrdU + nucleus. Scale bar: 1 mm. (**f**) Quantification of BrdU + nuclei in the dentate gyrus of Sham and PinX animals. Results are expressed as the mean number of BrdU + cells from 5 sections. Error bars represent mean ± SEM (n = 5 animals per group). **p < 0.005. (**g**) Representative schematic drawings of SVZ sections from sham-operated (sham, upper panel) and pinealectomized animals (PinX, lower panel). For each condition, anterior and posterior sections are shown. Each dot represents one BrdU + nucleus. LV = lateral ventricle. Scale bar: 1 mm. (**h**) Quantification of BrdU + nuclei in the SVZ of sham-operated and PinX animals. Results are expressed as the mean number of BrdU + cells from 6 sections. Error bars represent mean ± SEM (n = 5 animals per group). (**i**) Concentration of melatonin in blood plasma from sham-operated (open circles) and PinX animals (full circles) 7 days before (D-7), 7 and 30 days (D + 7 and D + 30) after surgery. Results are expressed in pg/ml ± SEM (n = 5 animals per group).

To determine whether photoperiod influences cell proliferation through a mechanism involving the pineal gland, i.v. BrdU was administered to pinealectomized (PinX) and to sham-operated animals (Sham) during MSP, at a time point corresponding to the peak of hypothalamic cell proliferation. The mean number of BrdU positive nuclei was estimated 24 h after BrdU injection in four hypothalamic slices per animal (n = 6 animals). As illustrated on representative schematic drawings (Fig. 4a) showing the spatial distribution of BrdU-positive nuclei in the hypothalami of ewes, a lower number of BrdU + nuclei was detected in PinX than in sham-operated animals (Fig. 4b; 69.0 ± 16.5 BrdU + nuclei and 279.4 ± 68.3 BrdU + nuclei in hypothalamic of PinX and Sham ewes respectively; p < 0.05). Western blot analysis also showed a significantly lower ratio for Cyclin D1/vinculin expression, indicating a marked decrease in Cyclin D1 expression in PinX compared to sham-operated animals (Fig. 4c,d, p < 0.001).

In the SGZ, a significant decrease in cell proliferation was observed in PinX animals (Fig. 4e) when quantifying the mean number of BrdU positive nuclei in five slices per animal (n = 6 animals; Fig. 4f; 27.5 ± 4.9 BrdU + nuclei and 73.4 ± 7.0 BrdU + nuclei in the SGZ of PinX and sham ewes respectively; p < 0.01), indicating that hippocampal cell proliferative activity was also affected by pinealectomy. In the SVZ, there was no effect of pinealectomy on the mean number of BrdU positive nuclei quantified in six slices per animal (n = 6 animals; Fig. 4g and h; 361.2 ± 33.1 BrdU + nuclei and 325.8 ± 25.3 BrdU + nuclei in the SVZ of PinX and Sham ewes respectively).

The concentration of nocturnal plasma melatonin was measured in both groups of animals (Sham and PinX)^8^. We found that melatonin concentration dropped below the detection level in PinX animals, 7 and 30 days following surgery (Fig. 4i; D-7: 157.7 ± 51.2 pg/ml; D + 7: Not detected; D + 30: Not detected), whereas melatonin concentrations remained stable before and after surgery in Sham animals (D-7: 126.4 ± 23.5 pg/ml; D + 7: 119.9 ± 51.7 pg/ml; D + 30: 113.4 ± 57.2 pg/ml).

### Neurogenesis impairment alters the seasonal timing of reproduction

**Figure 5:**
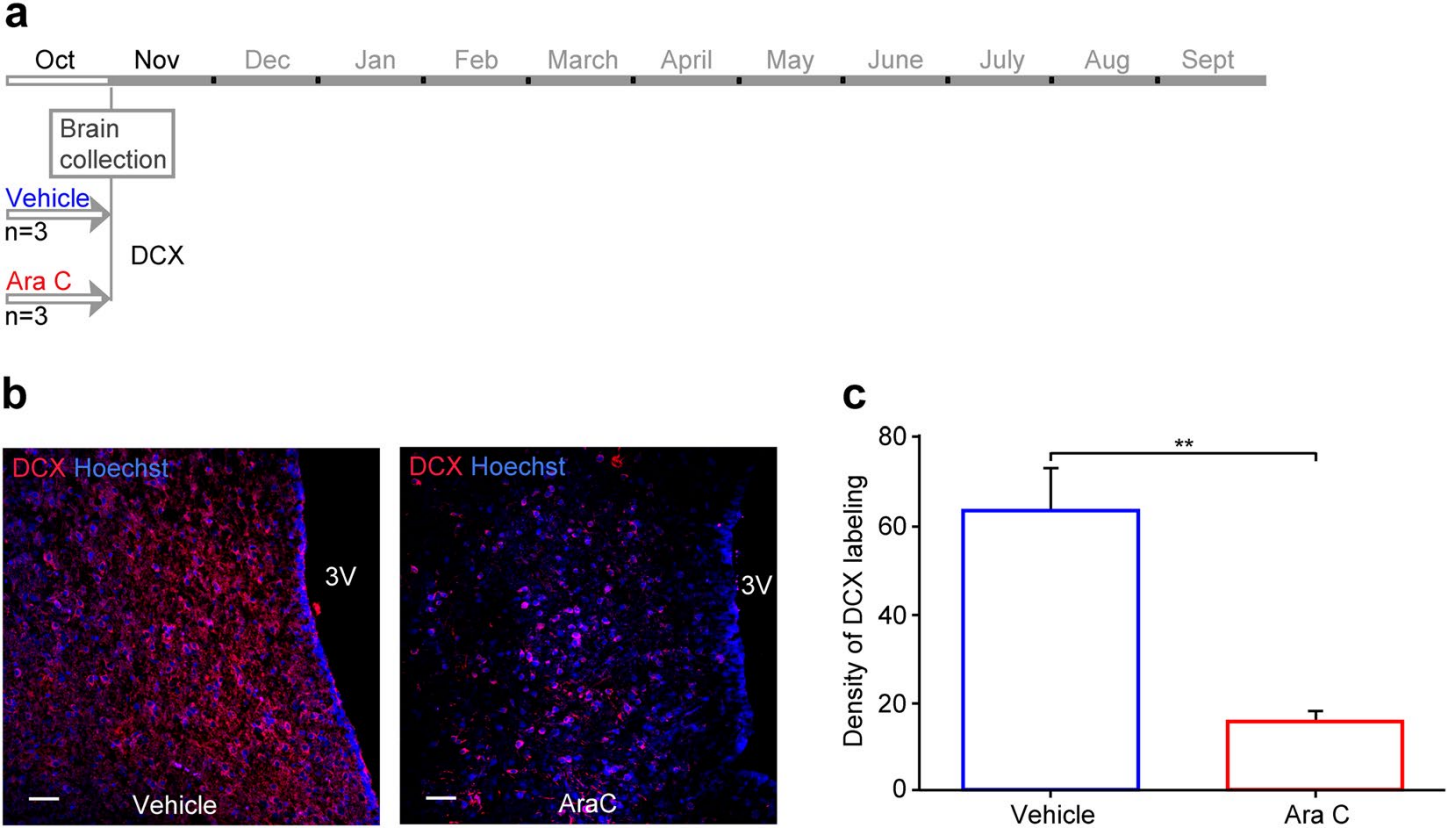
The administration of an antimitotic, Ara-C, alters hypothalamic neurogenesis. (**a**) Schedule of the experiment on hypothalamic neurogenesis: six ewes underwent a treatment with either vehicle (blue lines, n = 3) or the Ara-C (cytosine-b-D-arabinofuranoside) antimitotic drug, (red lines, n = 3), for 30 days during October corresponding to the MSP. Five days after the end of the treatments, brains were collected and assessed for neurogenesis. (**b**) Doublecortin (DCX, red) was used as a marker to assess neurogenesis rates as shown in fluorescence images of hypothalamic sections. Confocal images of DCX immuno-positive cells in hypothalamic brain regions from vehicle (left panel) and Ara-C (right panel) treated animals. Sections were counterstained with Hoechst (blue). 3 V = third ventricle. Scale bar: 50 µm. (**c**) Densities of DCX-positive cells in vehicle (blue lines) and Ara-C (red lines) treated animals. Results are presented as the number of DCX-positive cells per mm^2^. **P < 0.01.

To investigate the functional role of the hypothalamic cell proliferation/neurogenesis process, Ara-C (cytosine-b-D-arabinofuranoside), an antimitotic drug, was centrally administered to block neural progenitor cell division^25^ at 500 µg/day, a dose within the range of those used in i.c.v. treatment of mice and cats^26–28^. In one set of experiments, at a time point corresponding to the peak of hypothalamic neurogenesis activity (MSP), Ara-C was i.c.v. infused to a first group of ewes (n = 3), whereas a second group of ewes received vehicle (n = 3) for 30 days (Fig. 5a). At the end of this treatment, neurogenesis was asessed through DCX immunohistochemical detection. Figure 5b shows that fewer newborn neurons were observed in the Ara-C treated group than in the vehicle group. After quantification, significantly fewer DCX-positive cells were detected in animals treated with Ara-C (Fig. 5c, 64.9 ± 9.7 DCX + cells per mm² and 15.6 ± 4.2 DCX + cells per mm², respectively; p < 0.01) thus, validating the effect of Ara-C to block neurogenesis in the adult sheep hypothalamus.

**Figure 6:**
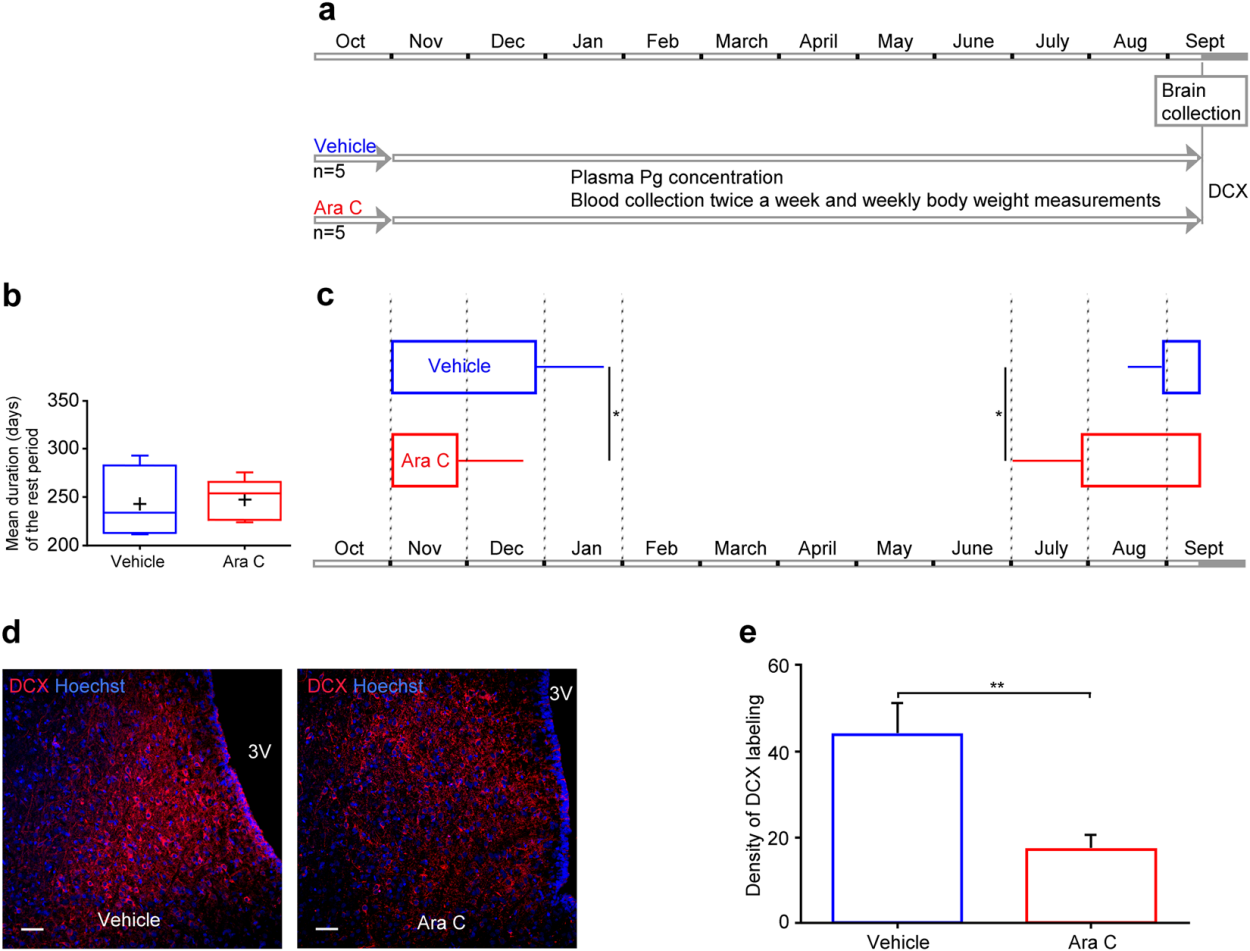
Ara-C administration alters reproductive programming. (**a**) Schedule of the experiment on reproductive programming: 10 ewes underwent a treatment with either vehicle (blue lines, n = 5) or the Ara-C (cytosine-b-D-arabinofuranoside) antimitotic drug (red lines, n = 5), for 30 days during October corresponding to the MSP. Cyclic activity was assessed through plasma progesterone concentration assayed twice weekly. The day after the end of the treatments, brains were collected and assessed for neurogenesis. (**b**) Mean ( ± SEM) duration (days) of the rest period in vehicle (blue lines) and Ara-C (red lines) treated animals. (**c**) Mean ( ± SEM) dates of the end of ovulatory activity followed by the mean ( ± SEM) dates of resuming ovulatory activity in vehicle (blue lines) and Ara-C (red lines) treated animals. (**d**) DCX (red) was used to assess the neurogenesis rates as shown in fluorescence images of hypothalamic sections. Confocal images of DCX immuno-positive cells in hypothalamic brain regions from vehicle (left panel) and Ara-C (right panel) treated animals. Sections were counterstained with Hoechst (blue). 3 V = third ventricle. Scale bar: 50 µm. (**e**) Densities of DCX-positive cells in vehicle (blue lines) and Ara-C (red lines) treated animals. Results are presented as the number of DCX-positive cells per mm^2^. **P < 0.01.

Our next aim was to investigate the effect of this decrease in neurogenesis on seasonal reproductive physiology. In a second set of experiments, ewes were infused with Ara-C (500 µg/day) and/or vehicle into the third ventricle for 30 days, blood samples were collected from the jugular vein twice weekly for 11 months, and plasma progesterone concentration was measured to assess seasonal changes in ovarian activity (Fig. 6a). There was no significant difference in body weight between control and treated ewes (Supplemental Material Figure S1). Although the length of the RP did not differ between the two groups (244.3 ± 18.9 days and 247.6 ± 9.6 days for the control and treated groups respectively; Fig. 6b), the mean date of the end of the breeding season (last elevated progesterone concentration) was significantly different between the vehicle and the Ara-C treated groups (Fig. 6c; p < 0.05). The breeding season finished significantly earlier in Ara-C treated than control group ewes (24 Nov ± 21.4 days and 28 Dec ± 17.8 days for the treated and control groups respectively; p < 0.05). Moreover, the date of onset of the following SP (first elevated progesterone concentration) was also significantly different between the two groups. The Ara-C-treated group started their breeding season significantly earlier than the control group (Fig. 6c; 29 Jul ± 26.7 days and 31 Oct ± 9.4 days the treated and control groups respectively; p < 0.05). At the end of the experiment there were significantly fewer DCX-positive cells in Ara-C treated animals than in controls (Fig. 4g and h, 43.6 ± 7.3 DCX + cells per mm² and 16.9 ± 3.6 DCX + cells per mm², respectively p < 0.01).

## Discussion

This longitudinal study highlighted an increase in hypothalamic cell proliferation occurring as a single peak during the middle of the SP and demonstrated that this enhancement was pineal dependent. Cell proliferation in the SGZ was found to be dependent of the time of the year and was also affected by pinealectomy. In contrast, cell proliferation in the SVZ was independent of the time of the year and insensitive to pinealectomy, endorsing the lack of photoperiod sensitivity of this neurogenic found in our previous study^17^.

The way hypothalamic neurogenesis relates functionally to seasonal reproductive physiology was assessed following a treatment with Ara-C, an antimitotic which decreases hypothalamic new neurons production. In treated animals, the timing for seasonal reproduction was altered. Overall, these results support the hypothesis that the pineal-dependent increase of hypothalamic neurogenesis is needed for appropriate timing of seasonal reproduction in sheep.

In the hypothalamus, a peak in proliferative activity was observed in the middle of the SP. This pattern of proliferative activity was detected both by BrdU incorporation and expression of Cyclin D1. The latter was used as a marker for proliferative activity because Cyclins D belong to a family of proteins responsible for regulating cell cycles and which promote the transition from the mid-G1 to the S-phase of the cell cycle^29^. The fact that there was a seasonal change in cell proliferation in the hypothalamus but not in the SVZ agrees well with data obtained previously on another cohort of ewes^17^. To our knowledge, the current research is the first longitudinal sex-specific study in a seasonal mammal exposed to natural variation of photoperiodic conditions reporting a seasonal change in cell proliferation. Using two quantifying methods, namely BrdU incorporation and Cyclin D1 expression, we demonstrated an increase in cell proliferation in the middle of both SP and RP in the hypothalamus and the hippocampal dentate gyrus, respectively. Recently, one study performed on Soay rams, another breed of sheep, showed that the level of cell proliferation was similar in these two structures^30^. Higher levels of cell proliferation were observed in the hypothalamus and the hippocampus during decreasing and increasing day length respectively. Overall, these data suggest that seasonal regulation of cell proliferation occurs in both sexes and in various breeds of sheep.

Initially identified in the avian brain^31–33^, photoperiodic fluctuations in cell proliferation and neurogenesis have also been reported in wild populations of voles (*Microtus pennsylvanicus*)^20,21^, golden hamsters^23^, white-footed mice (*Peromyscus leucopus*) and in Richardson’s ground squirrels (*Urocitellus richardsonii*^34^), while such seasonal effects were not detected in grey squirrels^35^. The photoperiodic regulation of cell proliferation, cell survival and neurogenesis may somehow vary depending on species and sex in these seasonal species^6,21,36^. Nevertheless, taken together with the aforementioned studies, the present data provide further evidence that a single environmental signal, namely photoperiod, can alter neurogenesis in adult mammals and suggest that adaptive responses to changing day length affect brain morphology and function.

In the present study, we showed that adult ewes deprived of seasonal time cues by pinealectomy had a significantly lower number of BrdU + cells in the hypothalamic and hippocampal neurogenic niches. Notably, pinealectomy was performed during the decreasing day length of autumn, a period where pineal exeresis has no effect on reproductive activity^37,38^. Our findings strongly suggest that pineal melatonin regulates the cell proliferation process and thus, in accordance with its direct modulatory effect on neural stem cell proliferation, differentiation and survival (for a review see^39^). Interestingly, *in vitro* melatonin was found to induce proliferation of cultured NSCs derived from the adult rat ventral midbrain^40^ and hippocampus^41^, and also from the adult mouse SVZ^42^. Melatonin regulates the cell viability of NSCs from different regions of the brain, including the rat midbrain^40^, and promotes survival of precursor cells *in vitro*. Recent *in vivo* studies have also reported that melatonin increases proliferative activity in the dentate gyrus of juvenil rats^43^ and promotes neurogenesis in the hippocampus of C57BL/6 mice^44,45^ and ovariectomized 129 Sv/EV mice^46^. Furthermore, the antidepressant agomelatine, which displays properties of a melatonin agonist, promotes neurogenesis in the rat dentate gyrus^47^. Thus, this indolamine appears as a likely candidate for a hormonal regulator of hypothalamic cell regeneration in seasonal mammals. However additional experiments will be necessary to define the role and the molecular mechanisms underlying the action of melatonin in regulating hypothalamic cell proliferation and neurogenesis in the ewe. One hypothesis is that melatonin effects could be mediated by neurotrophic factors, especially BDNF and GDNF, since melatonin has been found to increase these factors in cultured NSCs^40,48^.

In the adult rodent hypothalamus, the origin of the neurogenic potential is still a matter of debate. At least, two cell populations, namely the tanycytic neural stem cells and the NG2-glia, both targeted by the antimitotic drug Ara-C, show this property. Tanycytes are ependymoglial cells lining the third ventricular wall which retain the features of the radial glia cells^49^. Tanycytes, although showing a low frequency dividing rate^50,51^, have neurogenic and gliogenic properties *in vivo*^52–54^. The NG2-glia, a parenchymal population belonging to the oligodendrocyte cell line^55^ is able to proliferate and give rise to a small population of neurons that ultimately mature and integrate functional circuits^56,57^. Our results support the hypothesis of the involvement of tanycytic neural stem cells rather than the NG2-glia in the seasonal programming of the reproduction in sheep. Indeed, in our experience the administration of Ara-C has a duration of 30 days, compatible with the low rate of division of these NSCs. In addition, the implication of the NG2-glia population is likely very limited in time and amplitude mainly for two reasons. Firstly, in a recent study in mice, the infusion of Ara-C in the 3rd ventricle led to overfeeding and weight gain compared to controls within days following infusion, providing evidence for the role of NG2-glia in homeostasis via the down-regulation of leptin signaling^58^. In our study, no difference in body weight was detected between Ara-C-treated and control animals throughout the experiment, suggesting that the NG2-glia cell population was not involved. Second, the NG2-glia population was shown to have striking immediate abilities to self- renew *in vivo* as demonstrated in a genetically engineered NG2-glia traced mouse line. The almost complete depletion of the NG2-glia induced after an i.c.v. administration of Ara-C, was followed by a complete repopulation within 2 weeks^56^. In our study, the administration of Ara-C had a long-term effect on the sheep seasonal breeding programming at a time scale well above the fast regeneration capacity of NG2 cells (one month and up to 9 months, Fig. 6c), suggesting that this effect is based on new cabling of the neuronal circuits of reproduction depending on a robust neurogenesis process.

Recent data on the significance of hypothalamic neurogenesis have concluded that neurogenesis in the murine hypothalamus is crucial for controlling weight and metabolism^26,59^. In this study, we found that silencing neurogenesis in the adult ewe hypothalamus following an i.c.v. Ara-C treatment induced an earlier transition between the period of sexual activity and sexual rest, indicating that this process is involved in the timing of seasonal reproduction likely through a melatonin dependent effect. Our data showed a single peak of proliferative activity occurring in the middle of the sexual period (MSP) which is approximately 3 months before the transition between the sexual and the rest period^19^. In contrast to rodent species, neuronal maturation is a much longer process in the ovine brain. At least in the main olfactory bulb (MOB), newly generated neurons do not differentiate until 90 days of age^60^. With respect to a comparable maturation time, the new hypothalamic neurons born during MSP could then be functionally mature in time for the transition between the active and the resting reproductive state in this species that occurs at the end of January and could play a functional role in this transition.

The molecular and cellular mechanisms underlying this outcome are under investigation, nevertheless in light of these data, a possible explanation is that in Ara-C treated animals the decrease in neurogenesis leads to the GnRH pulse generator being deactivated faster via a possible effect on RF-amide, arginine-phenylalanine-amide (RFRP) neurons. These neurons are presumed to be involved in the transitions between reproductive and non –reproductive states^61^. The available data indeed show that RFRP3 (RF-amide–related peptide-3), a member of the RF-amide family of peptides whose expression is melatonin-dependent^61,62^, inhibits LH pulsatility and blocks the LH surge induced by estradiol benzoate in ovariectomized animals^63,64^. In addition, the use of a RFRP3 receptor antagonist, RF9, caused a strong increase in LH plasma concentrations^65^. In contrast, the duration of the RP appears to be regulated by different mechanisms, since this paradigm was not affected by the Ara-C treatment.

In conclusion, our study demonstrates a seasonal pattern of cell proliferation in the adult sheep hypothalamus and our *in vivo* data support the hypothesis that melatonin is a potential hormonal regulator for this seasonal increase. Furthermore, we provide the first *in vivo* investigation of the role of adult neurogenesis in the sheep hypothalamus that strongly supports its role in the seasonal synchronization of reproduction. Future work is required to identify which pre-existing synaptic circuits can incorporate new hypothalamic neurons. Like in songbirds, where correlations between neuronal replacement and the changing environment at different times of the year suggest that adult neurogenesis is linked to circuit plasticity^31,66^, our findings bring to light new perspectives on the functional role of adult hypothalamic neurogenesis as an elemental mechanism by which mammals adapt to the changing environment.

## Materials and Methods

### Animals

This study was approved by the Val de Loire animal experimentation ethics committee (CEEAVdL) and was in accordance with the guidelines of the French Ministry of Agriculture for animal experimentation and European regulations on animal experimentation (2010/63/EU). Experiments were performed in accordance with regulations regarding animals (authorization N° 006352 of the French Ministry of Agriculture in accordance with EEC directive). All experiments were performed in adult Ile-de-France (IF) ewes gathered under standard husbandry at the INRA Val-de-Loire research center (Nouzilly, Indre-et-Loire, France, 47°33′00.8“N 0°46′55.3“E). The experimental facilities are approved by the local authority (agreement number E37–175–2). The study animals were 2–3-year old primiparous ewes. They were fed daily with hay and pellets and had access to water *ad libitum*. Ewes were exposed to the natural photoperiod throughout the experiment and were allowed to move in all part of the livestock building. The experimental building was isolated from the rest of the herd and caution was payed to avoid any exposition of the ewes to the male odor which could interfere with the timing of the breeding season. In these conditions, the locomotor activity of all the animals was the same throughout the experiments.

### BrdU treatments

For the proliferation studies, a single i.v. injection of 20 mg/kg of BrdU (Sigma-Aldrich, Saint-Quentin Fallavier, France) was administered between 09:00 and 10:30 a.m.^17^. Ewes were euthanized 24 hours after the BrdU injection.

### Proliferation kinetics

Variation in hypothalamic proliferation throughout the year was studied in 30 ewes (n = 15; 55.3 ± 5.5 kg and n = 15, 58.5 ± 8.7 kg body weight for the sexual activity period and for the sexual rest period respectively). The BrdU labelled nuclei were counted at six different time points in the year according to the seasonal cyclic activity of the ewes. To that end, three groups of five ewes were formed at different times in the sexual activity period (SP) which starts in August in the IF breed^19^, at the onset (OSP), in the middle (MSP) and at the end (ESP) of the sexual activity period. Another three groups of five ewes were constituted during the sexual rest period (RP) that starts in mid-January in the IF breed, at the onset (ORP), in the middle (MRP) and at the end (ERP) of the sexual rest period. These six experimental groups were formed according to plasma progesterone levels which were evaluated for each ewe from blood samples collected twice weekly. With these progesterone assays the onset and end of the sexual activity period were determined. The time of the BrdU injection for each group is detailed in Fig. 1a. All ewes were euthanized 24 hours after BrdU injection. Brains were fixed and sectioned as described below. For this experiment, ten series of five hypothalamic coronal sections (10 µm) were collected. Between each series, sections that were not collected for immunohistochemistry were transferred into a tube and kept at −20 °C until western blot analysis.

## Effect of Pinealectomy

Surgery: Five ewes underwent pinealectomy (PinX, 56.2 ± 6.6 kg BW) and five ewes were sham operated (Sham, 63.2 ± 5.2 kg BW) at the CIRE platform (Veterinary surgery and imaging platform for research and education, INRA Centre of Tours^67,68^). Details are described in Supplemental Material Part1.

Melatonin assays: The animals were operated on during estrus whose onset was determined by taking two blood samples per week. Seven days before surgery, blood samples were collected during the night, two hours after sunset and then every hours for 4 hours. After surgery at D + 7 and D + 30, blood samples were collected in the middle of the night to check that surgery had been successful and the level of melatonin secretion in sham-operated animals (Fig. 3i). Blood plasma was stored at -20 °C until assay. Plasma melatonin concentration was determined in 100 µL aliquots using a well-validated radio immune-assay^69^ (RIA). Standards were prepared in plasma and assay sensitivity averaged 4 pg/mL for a 100 µL plasma sample. Intra- and inter-assay CVs were 7.2% and 11.2% respectively.

To assess cell proliferation, two months after surgery the ewes received a single BrdU injection and were euthanized 24 hours later. Brains were fixed and sectioned. As in the first experiment, hypothalamic coronal sections (10 µm) were collected and treated for immunohistochemistry following the experimental protocol described above or conserved at -20 °C until western blot analysis.

### Antimitotic drug Ara-C (cytosine-b-D-arabinofuranoside) treatment

At the beginning of October, 16 ewes (n = 8 vehicle, 58.8 ± 4.5 kg BW and n = 8 AraC, 59.4 ± 4 kg BW) were implanted with a stainless-steel guide cannula in the third ventricle (3 V) of the brain under general isoflurane anesthesia, in accordance with the method previously described^67,70^. Briefly, radio-opaque material (OmnipaqueR; Nycomed Ingenon SA, Paris, France) was injected through a cannula into the lateral ventricle delimiting the ventricular system, more specifically the 3 V. The cannula (length 55 mm; outer diameter 1.5 mm; inner diameter 0.8 mm) was inserted into the 3 V and the outflow of CSF through the cannula was checked when in place. The cannula was then connected to a subcutaneous osmotic pump (Alzet osmotic pump 2ML4 model). The pump rate was 2.5 µL/hour for four weeks. Half of the ewes received saline solution (n = 8, vehicle) and the other half received AraC (n = 8), at 500 µg/day, a dose within the range of those used in i.c.v. treatment of mice and cats^26–28^. After four weeks, the pumps were removed and three ewes from each group were euthanized, their hypothalami were handled for DCX immunofluorescence. The remaining ewes were kept alive to study their ovulatory activity. During the next 11 months two blood samples per week were collected to assess the plama progesterone concentration in order to determine when the sexual activity period ended and the following started. Ewes were weighed every week. At the end of this period, the ewes were euthanized and their hypothalami handled for DCX immunofluorescence.

### Tissue preparation

At the end of each experiment all the ewes were euthanized with an overdose of sodium pentobarbitone (25 mg/kg; Merial, Lyon, France) preceded by an injection of heparin (25,000 IU, i.v.). After euthanasia, animals were decapitated and the heads were perfused through both carotid arteries with 2 L sodium nitrite (1%) in NaCl (0.9%) followed by 4 L of cold paraformaldehyde (4%) in 0.1 M phosphate buffer, pH 7.4^71^. Three blocks of brain encompassing the hypothalamic, the SVZ and the dorsal hippocampal structures were collected. After a 48-hour period of postfixation, blocks were soaked in 20% sucrose for cryoprotection. The blocks of brains were then processed for immunohistochemistry as detailed below.

## Histological procedures and immunohistochemistry

### Cryostat sectioning

Immediately prior to sectioning, the brain blocks were frozen by immersion in nitrogen-cooled isopentane. Coronal sections were processed and mounted directly onto SUPERFROST PLUS (Fisher Scientific, Illkirch, France) slides and kept at −80 °C^16,17^.

Hypothalamic coronal sections (10 µm) were collected from the premammillary recess over a 2.5-mm rostral direction. Hippocampus coronal sections (30 µm) from the dorsal hippocampus were collected from the beginning of the dentate gyrus for a 4 mm in a caudal direction. SVZ coronal sections (10 µm) were collected from the opening of the lateral ventricles for a 2.5 mm in a caudal direction.

### Immunohistochemical protocol

Immunohistochemical reactions were carried out using single immunoperoxidase (BrdU^17^) or immunofluorescence (DCX-Hoechst^16^) methods on cryostat sections incubated overnight at room temperature with primary antibodies (BrdU, rat monoclonal, AbCys, AbC117-7513, 1/250, DCX Santa Cruz Biotechnology, goat polyclonal, sc8066, 1/1200) and for 90 minutes at room temperature with secondary antibodies (Donkey anti-rat/Po, Jackson immunoresearch, 712-035-153, Donkey anti-goat/Alexa555, Molecular Probes, A21432). Single staining using peroxidase was performed with peroxidase-conjugated secondary antibodies detected with 3,3′diaminobenzidine (DAB) in 50 mM Tris-HCl (pH 7.6) containing 0.025% hydrogen peroxide for 10 minutes followed by washing with 50 mM Tris-HCl (pH 7.6). Antibodies were diluted in Tris-buffered saline (TBS; pH 7.4) containing 0.3% Triton and 0.2% of normal horse serum. BrdU and DCX immunohistochemistry included an initial step to unmask and denature endogenous DNA using 2 N HCl for 30 minutes at room temperature. Sections were incubated in blocking buffer (TBS, 0.3% triton and 2% normal horse serum) for 30 minutes at room temperature. Staining with neutral red was performed to visualize the shape and size of the cell nuclei close to the BrdU-labeled cells and to define the outline of the SVZ and DG.

### Quantification of immunolabeled cells

The BrdU + nuclei were quantified using a light microscope (Axioskope 2, Zeiss, Germany) at a magnification of x20 and cell count analysis software (computerized image analysis Mercator, Explora Nova, La Rochelle, France). BrdU + cell counts were represented as median and interquartiles ranges. The hypothalamic area used for counting included 4 mm laterally on each side of the third ventricular walls and up to the upper limit of the third ventricle dorsally. Four sections separated by 600 µm were processed for BrdU immunoperoxidase or for DCX immunofluorescence.

The SVZ outline was delimited with neutral red coloration with an x10 objective. Considering the large size of the neurogenic areas in the SVZ and the hippocampal DG, two series separated by 2.5-mm of anterior and posterior slices were quantified. In each series, three sections separated by 80 µm were processed for BrdU immunoperoxidase. The DG outline was delimited in the same way. Five sections separated by 800 µm were processed for BrdU immunoperoxidase. For both SVZ and DG no significant differences were detected between the anterior and the posterior labeling (data not shown), therefore both series of data were pooled.

To count DCX-positive cells and assess their distribution, hypothalamic frontal sections were analyzed using a Zeiss Axioscop 2 microscope equipped with a motorized X–Y-sensitive stage and a fluorescent lamp under a magnification of 109 coupled to a video CDD camera connected to a computerized image analysis system (Explora Nova, La Rochelle, France). Images were acquired using Mercator software (Explora Nova, La Rochelle, France) by applying the same contrast and exposition time for acquisition to compare labeled series accuratly. Cell nuclei were labeled with Hoechst (33258, Molecular Probes, Eugene, OR) to ensure cells were labeled accurately. The images were created in tiff files and included in the figures using Adobe Photoshop (San Jose, CA). The results are expressed as the density of DCX + cells per mm^2^.

DCX + cells were quantified in the arcuate nucleus, within two rectangular areas (200 µm x 300 µm) positioned on each side of the third ventricle. The counter was blind to the experimental group.

#### Western blotting experiments

Proteins from brain sections were extracted as previously described^72^ and adapted for fixed tissues^73^. Lysates were centrifuged at 12000 g for 30 min at 4 °C, and the protein concentration was determined using a BCA protein assay. Protein extracts were denatured and samples underwent electrophoresis on 12% SDS-polyacrylamide gel and were transferred onto nitrocellulose membranes. Blots were blocked with Tris-buffered saline buffer supplemented with 0.1% Tween 20 and 5% milk for 30 min at room temperature and membranes were then incubated overnight at 4 °C with specific rabbit antibodies against cyclin D1 (Santa Cruz Biotechnology, Texas, USA) or mouse antibodies against vinculin used as an internal standard (Sigma, St. Louis, MO, USA) at 1/1,000 final dilution. Blots were washed several times and incubated at room temperature for 1h30 with a HRP-conjugated anti-rabbit or anti-mouse IgG (dilution 1/5,000). The signal was detected with an ECL chemiluminescence kit using a G:Box SynGene apparatus (Ozyme) with GeneSnap software (release 4.01.02). The results are expressed as the signal intensity in arbitrary units after normalizing with the presence of vinculin. No western blot analysis could be performed with the ORP samples because of technical problems (frozen samples were accidentally thawed).

#### Statistical analyses

Normal distribution and equal variance assumptions were tested using the Shapiro-Wilk normality and Bartlett’s tests, respectively. Multivariable datasets were analyzed using ANOVA, followed by a Bonferroni’s multiple comparison test to produce multiplicity-adjusted p values for pairwise comparisons. Significance was defined as a p value of 0.05 or smaller. Single variable pairwise comparisons were analyzed using a Student’s t test.

## Electronic supplementary material

Supplementary Information

## References

[1] Hazlerigg, D. & Simonneaux, V. In *Physiology of reproduction* Vol. 2 (eds Knobil & Neill) 1575–1604 (Academic press, 2014).

[2] Reiter RJ (1991). Pineal melatonin: cell biology of its synthesis and of its physiological interactions. Endocr Rev.

[3] Bartness TJ, Powers JB, Hastings MH, Bittman EL, Goldman BD (1993). The timed infusion paradigm for melatonin delivery: what has it taught us about the melatonin signal, its reception, and the photoperiodic control of seasonal responses?. Journal of pineal research.

[4] Goldman BD (2001). Mammalian photoperiodic system: formal properties and neuroendocrine mechanisms of photoperiodic time measurement. Journal of biological rhythms.

[5] Hiebert SM, Green SA, Yellon SM (2006). Daily timed melatonin feedings mimic effects of short days on testis regression and cortisol in circulation in Siberian hamsters. Gen Comp Endocrinol.

[6] Walton JC, Chen Z, Travers JB, Nelson RJ (2013). Exogenous melatonin reproduces the effects of short day lengths on hippocampal function in male white-footed mice, Peromyscus leucopus. Neuroscience.

[7] Reiter RJ (1980). The pineal and its hormones in the control of reproduction in mammals. Endocr Rev.

[8] Malpaux B, Migaud M, Tricoire H, Chemineau P (2001). Biology of mammalian photoperiodism and the critical role of the pineal gland and melatonin. Journal of biological rhythms.

[9] Zhao C, Deng W, Gage FH (2008). Mechanisms and functional implications of adult neurogenesis. Cell.

[10] Migaud M (2010). Emerging new sites for adult neurogenesis in the mammalian brain: a comparative study between the hypothalamus and the classical neurogenic zones. The European journal of neuroscience.

[11] Migaud M, Butrille L, Batailler M (2015). Seasonal regulation of structural plasticity and neurogenesis in the adult mammalian brain: focus on the sheep hypothalamus. Frontiers in neuroendocrinology.

[12] Lee DA, Blackshaw S (2012). Functional implications of hypothalamic neurogenesis in the adult mammalian brain. Int J Dev Neurosci.

[13] Rojczyk-Golebiewska E, Palasz A, Wiaderkiewicz R (2014). Hypothalamic subependymal niche: a novel site of the adult neurogenesis. Cell Mol Neurobiol.

[14] Kokoeva MV, Yin H, Flier JS (2007). Evidence for constitutive neural cell proliferation in the adult murine hypothalamus. The Journal of comparative neurology.

[15] McNay DE, Briançon N, Kokoeva MV, Maratos-Flier E, Flier JS (2012). Remodeling of the arcuate nucleus energy-balance circuit is inhibited in obese mice. J Clin Invest.

[16] Batailler M (2014). DCX-expressing cells in the vicinity of the hypothalamic neurogenic niche: a comparative study between mouse, sheep, and human tissues. J Comp Neurol.

[17] Migaud M, Batailler M, Pillon D, Franceschini I, Malpaux B (2011). Seasonal changes in cell proliferation in the adult sheep brain and pars tuberalis. Journal of biological rhythms.

[18] Batailler M, Derouet L, Butruille L, Migaud M (2016). Sensitivity to the photoperiod and potential migratory features of neuroblasts in the adult sheep hypothalamus. Brain structure & function.

[19] Chanvallon A (2011). New insights into the influence of breed and time of the year on the response of ewes to the ‘ram effect’. Animal: an international journal of animal bioscience.

[20] Galea LA, McEwen BS (1999). Sex and seasonal differences in the rate of cell proliferation in the dentate gyrus of adult wild meadow voles. Neuroscience.

[21] Ormerod BK, Galea LA (2003). Reproductive status influences the survival of new cells in the dentate gyrus of adult male meadow voles. Neuroscience letters.

[22] Bartkowska K, Djavadian RL, Taylor JR, Turlejski K (2008). Generation recruitment and death of brain cells throughout the life cycle of Sorex shrews (Lipotyphla). Eur J Neurosci.

[23] Huang L, DeVries GJ, Bittman EL (1998). Photoperiod regulates neuronal bromodeoxyuridine labeling in the brain of a seasonally breeding mammal. Journal of neurobiology.

[24] Smith MT, Pencea V, Wang Z, Luskin MB, Insel TR (2001). Increased number of BrdU-labeled neurons in the rostral migratory stream of the estrous prairie vole. Hormones and behavior.

[25] Doetsch F, Caille I, Lim DA, Garcia-Verdugo JM, Alvarez-Buylla A (1999). Subventricular zone astrocytes are neural stem cells in the adult mammalian brain. Cell.

[26] Kokoeva MV, Yin H, Flier JS (2005). Neurogenesis in the hypothalamus of adult mice: potential role in energy balance. Science.

[27] Breton-Provencher V, Lemasson M, Peralta MR, Saghatelyan A (2009). Interneurons produced in adulthood are required for the normal functioning of the olfactory bulb network and for the execution of selected olfactory behaviors. J Neurosci.

[28] Dutheil S, Brezun JM, Leonard J, Lacour M, Tighilet B (2009). Neurogenesis and astrogenesis contribution to recovery of vestibular functions in the adult cat following unilateral vestibular neurectomy: cellular and behavioral evidence. Neuroscience.

[29] Kozar K (2004). Mouse development and cell proliferation in the absence of D-cyclins. Cell.

[30] Hazlerigg DG, Wyse CA, Dardente H, Hanon EA, Lincoln GA (2013). Photoperiodic variation in CD45-positive cells and cell proliferation in the mediobasal hypothalamus of the Soay sheep. Chronobiol Int.

[31] Nottebohm F (1981). A brain for all seasons: cyclical anatomical changes in song control nuclei of the canary brain. Science.

[32] Paton JA, Nottebohm FN (1984). Neurons generated in the adult brain are recruited into functional circuits. Science.

[33] Sherry DF, Hoshooley JS (2010). Seasonal hippocampal plasticity in food-storing birds. Philosophical transactions of the Royal Society of London. Series B, Biological sciences.

[34] Burger DK, Saucier JM, Iwaniuk AN (2013). & Saucier DM. Seasonal and sex differences in the hippocampus of a wild rodent. Behav Brain Res.

[35] Lavenex P, Steele MA, Jacobs LF (2000). The seasonal pattern of cell proliferation and neuron number in the dentate gyrus of wild adult eastern grey squirrels. Eur J Neurosci.

[36] Walton JC, Pyter LM, Weil ZM, Nelson RJ (2012). Photoperiod mediated changes in olfactory bulb neurogenesis and olfactory behavior in male white-footed mice (Peromyscus leucopus). PLoS One.

[37] Wayne NL, Malpaux B, Karsch FJ (1990). Photoperiodic requirements for timing onset and duration of the breeding season of the ewe: synchronization of an endogenous rhythm of reproduction. Journal of comparative physiology. A, Sensory, neural, and behavioral physiology.

[38] Woodfill CJ, Wayne NL, Moenter SM, Karsch FJ (1994). Photoperiodic synchronization of a circannual reproductive rhythm in sheep: identification of season-specific time cues. Biology of reproduction.

[39] Chu J (2016). Effects of melatonin and its analogues on neural stem cells. Molecular and cellular endocrinology.

[40] Kong X (2008). Melatonin regulates the viability and differentiation of rat midbrain neural stem cells. Cell Mol Neurobiol.

[41] Tocharus C (2014). Melatonin enhances adult rat hippocampal progenitor cell proliferation via ERK signaling pathway through melatonin receptor. Neuroscience.

[42] Sotthibundhu A, Phansuwan-Pujito P, Govitrapong P (2010). Melatonin increases proliferation of cultured neural stem cells obtained from adult mouse subventricular zone. Journal of pineal research.

[43] Kim MJ, Kim HK, Kim BS, Yim SV (2004). Melatonin increases cell proliferation in the dentate gyrus of maternally separated rats. Journal of pineal research.

[44] Ramirez-Rodriguez G, Klempin F, Babu H, Benitez-King G, Kempermann G (2009). Melatonin modulates cell survival of new neurons in the hippocampus of adult mice. Neuropsychopharmacology.

[45] Ramirez-Rodriguez G, Ortiz-Lopez L, Dominguez-Alonso A, Benitez-King GA, Kempermann G (2011). Chronic treatment with melatonin stimulates dendrite maturation and complexity in adult hippocampal neurogenesis of mice. Journal of pineal research.

[46] Crupi R (2011). Melatonin’s stimulatory effect on adult hippocampal neurogenesis in mice persists after ovariectomy. Journal of pineal research.

[47] Banasr M, Soumier A, Hery M, Mocaer E, Daszuta A (2006). Agomelatine, a new antidepressant, induces regional changes in hippocampal neurogenesis. Biol Psychiatry.

[48] Niles LP (2004). Neural stem cells express melatonin receptors and neurotrophic factors: colocalization of the MT1 receptor with neuronal and glial markers. BMC Neurosci.

[49] Pérez-Martín M (2010). IGF-I stimulates neurogenesis in the hypothalamus of adult rats. Eur J Neurosci.

[50] Kokoeva MV, Yin H, Flier JS (2007). Evidence for constitutive neural cell proliferation in the adult murine hypothalamus. J Comp Neurol.

[51] Haan N (2013). Fgf10-expressing tanycytes add new neurons to the appetite/energy-balance regulating centers of the postnatal and adult hypothalamus. The Journal of neuroscience: the official journal of the Society for Neuroscience.

[52] Xu Y (2005). Neurogenesis in the ependymal layer of the adult rat 3rd ventricle. Experimental neurology.

[53] Lee DA (2012). Tanycytes of the hypothalamic median eminence form a diet-responsive neurogenic niche. Nature neuroscience.

[54] Robins, S. C. *et al*. α-Tanycytes of the adult hypothalamic third ventricle include distinct populations of FGF-responsive neural progenitors. *Nat Commun* **4** (2013). 10.1038/ncomms3049 23804023

[55] Dimou L, Simon C, Kirchhoff F, Takebayashi H, Gotz M (2008). Progeny of Olig2-expressing progenitors in the gray and white matter of the adult mouse cerebral cortex. J Neurosci.

[56] Robins SC (2013). Extensive regenerative plasticity among adult NG2-glia populations is exclusively based on self-renewal. Glia.

[57] Robins SC (2013). Evidence for NG2-glia derived, adult-born functional neurons in the hypothalamus. PLoS One.

[58] Djogo T (2016). Adult NG2-Glia Are Required for Median Eminence-Mediated Leptin Sensing and Body Weight Control. Cell metabolism.

[59] Lee DA (2014). Dietary and sex-specific factors regulate hypothalamic neurogenesis in young adult mice. Front Neurosci.

[60] Brus M (2013). Dynamics of olfactory and hippocampal neurogenesis in adult sheep. The Journal of comparative neurology.

[61] Klosen P, Sebert ME, Rasri K, Laran-Chich MP, Simonneaux V (2013). TSH restores a summer phenotype in photoinhibited mammals via the RF-amides RFRP3 and kisspeptin. FASEB journal: official publication of the Federation of American Societies for Experimental Biology.

[62] Ubuka T (2012). Identification, expression, and physiological functions of Siberian hamster gonadotropin-inhibitory hormone. Endocrinology.

[63] Clarke IJ (2008). Potent action of RFamide-related peptide-3 on pituitary gonadotropes indicative of a hypophysiotropic role in the negative regulation of gonadotropin secretion. Endocrinology.

[64] Smith JT (2008). Variation in kisspeptin and RFamide-related peptide (RFRP) expression and terminal connections to gonadotropin-releasing hormone neurons in the brain: a novel medium for seasonal breeding in the sheep. Endocrinology.

[65] Caraty A (2012). RF9 powerfully stimulates gonadotrophin secretion in the ewe: evidence for a seasonal threshold of sensitivity. J Neuroendocrinol.

[66] Nottebohm F (2004). The road we travelled: discovery, choreography, and significance of brain replaceable neurons. Ann N Y Acad Sci.

[67] Thiery JC (2006). Concentrations of estradiol in ewe cerebrospinal fluid are modulated by photoperiod through pineal-dependent mechanisms. Journal of pineal research.

[68] Cognié, J. & Migaud, M. In *Step by step experimental pinealectomy techniques in animals for researchers* (ed Turgut, M.) Ch. 7, 137–151 (NOVA Science Publishers, 2013).

[69] Malpaux B, Daveau A, Maurice F, Locatelli A, Thiery JC (1994). Evidence that melatonin binding sites in the pars tuberalis do not mediate the photoperiodic actions of melatonin on LH and prolactin secretion in ewes. Journal of reproduction and fertility.

[70] Skinner DC, Malpaux B, Delaleu B, Caraty A (1995). Luteinizing hormone (LH)-releasing hormone in third ventricular cerebrospinal fluid of the ewe: correlation with LH pulses and the LH surge. Endocrinology.

[71] Batailler M, Caraty A, Malpaux B, Tillet Y (2004). Neuroanatomical organization of gonadotropin-releasing hormone neurons during the oestrus cycle in the ewe. BMC neuroscience.

[72] Tosca L (2011). Metformin decreases GnRH- and activin-induced gonadotropin secretion in rat pituitary cells: potential involvement of adenosine 5′ monophosphate-activated protein kinase (PRKA). Biology of reproduction.

[73] Guo H (2012). An efficient procedure for protein extraction from formalin-fixed, paraffin-embedded tissues for reverse phase protein arrays. Proteome science.

