# Supplementary material for: Pineal-dependent increase of hypothalamic neurogenesis contributes to the timing of seasonal reproduction in sheep
Source: Sci Rep. 2018 Apr 18;8:6188. doi: 10.1038/s41598-018-24381-4 (PMC5906660; doi:10.1038/s41598-018-24381-4)
Supplement: Supplementary file 1 — Supplementary Information [file 41598_2018_24381_MOESM1_ESM.docx]

**Supplementary Information**

**Pineal-dependent increase of hypothalamic neurogenesis contributes to the timing of seasonal reproduction in sheep**

Martine Batailler^1^,

Didier Chesneau^1^,

Laura Derouet^1^,

Lucile Butruille^1^,

Stéphanie Segura^1^,

Juliette Cognié^1^,

Joëlle Dupont^1^,

Delphine Pillon^1^

Martine Migaud^1*^

1 : INRA, UMR 85 Physiologie de la Reproduction et des Comportements, F-37380 Nouzilly, France ; CNRS, UMR7247, F-37380 Nouzilly, France ; Université de Tours, F-37041 Tours, France ; Institut Français du Cheval et de l’Equitation (IFCE), F-37380 Nouzilly, France

* Corresponding author

**Table of Contents:**

**Supplemental Material Part1:** Surgical procedure for the pinealectomy

**Figure S1:** Body weight measurements of the vehicle and Ara-C treated ewes.

**Supplemental Material Part1:** Procedure for the pinealectomy.

Procedure for the pinealectomy :The animals were fasted 24h before surgery. Following premedication with an i.v. injection of thiopental (14mg/kg body weight, BW; Nesdonal, Merial, Villeurbanne, France), the animals were intubated and maintained under anesthesia with a closed circuit of 3% isofluorane (Vetflurane, Virbac, Carros, France) and 100% oxygen. A catheter fixed within the jugular vein was connected to a perfusion of physiological serum supplemented with morphine (0.5 mg/kg BW, Morphine, Lavoisier, Paris, France), lidocaine (2.4 mg/kg BW, Lurocaïne, Vetoquinol, Luré, France) and ketamine (0.8 mg/kg BW, Imalgène 1000, Merial, Villeurbanne, France), to ensure analgesia during the 2-hour surgery procedure. Animals were placed in a prone position with their heads fixed in a stereotaxic frame. The parietal skull was exposed and six holes (1 cm wide) were drilled into the skull forming a paramesial left rectangle (3.5x2.5 cm) exposing the midline. The dura mater was carefully punctured and cut laterally to the sagittal sinus, exposing the left parietooccipital cortex. Using a ribbon retractor, the occipital lobe was gently compressed and the veins from the cortex to the sagittal sinus were electrocoagulated. Dissection was conducted through delicate traction and the gland was freed from all the veins and emerged dorsally. Sham-operated animals were sutured at that point, whereas for PinX animals non-traumatic dissecting forceps were then used to grasp the gland. Dura mater was sutured and protected with a piece of polymer (Pangen®, Urgo, Chenôve, France). The hole through the skull was closed with acrylic resin (Meliodent Rapid Repair, Heraeus Kulzer GmbH, Hanau, Germany). Continuous subcutaneous sutures were carried out (Safil® 0, B/Braun, Tuttlingen, Germany) to cover this resin and the skin incision was sutured (Flexidene®2, B/Braun, Tuttlingen, Germany) with continuous stitching. Post-operative ventilation with oxygen was maintained until first signs of awakening appeared. Animals were then housed individually for 6 hours in a padded stall before being put back with congeners. They received an anti-inflammatory drug for 4 days (2 mg/kg BW flunixin meglumin, Finadyne®, Intervet, Beaucouzé, France), anti-edema medication: 1 mg/kg BW of furosemide (Dimazon®, Intervet, Beaucouzé, France) at the end of surgery and 3 mg/kg BW hydrochlorothiazin with 0.03 mg/kg BW dexamethason (Diurizone®, Vétoquinol, Luré, France) for 3 days and 0.5 mg/kg BW morphine every 6 hours for 24 hours post-surgery.

Supplemental Figure 1

**Figure S1:** Body weight measurements of the vehicle and Ara-C treated ewes. Mean (± SEM) body weight (BW) during a 8-months period (31 weeks) for vehicle (blue line) or AraC treated ewes (red line). Body weight was measured once a week from the 29^th^ of January up to the 27^th^ of August, during the sexual rest period. No significant difference in the mean BW between the two groups were found (ANOVA). Due to their canula implantation device, the ewes had to be housed in a sheepfold in which they benefited from natural light but were placed in individually boxes which limited their movements, consequently their physical exercice were reduced leading to an increase in BW for both groups.
